# Effects of Chitosan Molecular Weight and Degree of Deacetylation on Chitosan−Cellulose Nanocrystal Complexes and Their Formation

**DOI:** 10.3390/molecules28031361

**Published:** 2023-01-31

**Authors:** Hezhong Wang, Maren Roman

**Affiliations:** 1 Department of Sustainable Biomaterials, Virginia Tech, Blacksburg, VA 24061, USA; 2 Macromolecules Innovation Institute, Virginia Tech, Blacksburg, VA 24061, USA

**Keywords:** cellulose, chitosan, polysaccharide, polyelectrolyte, complexation, nanocrystals

## Abstract

This study was conducted to determine the effects of chitosan molecular weight and degree of deacetylation (DD) on chitosan–cellulose nanocrystal (CNC) polyelectrolyte–macroion complexes (PMCs) and their formation. Chitosan samples with three different molecular weights (81, 3 · 10^3^, 6 · 10^3^ kDa) and four different DDs (77, 80, 85, 89%) were used. The effects on PMC formation were determined by turbidimetric titration. An effect of the molecular weight of chitosan was not observed in turbidimetric titrations. Turbidity levels were higher for CNCs with lower sulfate group density and larger hydrodynamic diameter than for CNCs with higher sulfate group density and smaller hydrodynamic diameter. Conversely, turbidity levels were higher for chitosans with higher DD (higher charge density) than for chitosans with lower DD (lower charge density). PMC particles from chitosans with different molecular weights were characterized by scanning electron microscopy, laser Doppler electrophoresis, and dynamic light scattering. PMCs from high-molecular-weight chitosan were more spherical and those from medium-molecular-weight chitosan had a slightly larger hydrodynamic diameter than PMCs from the respective other two chitosans. The molecular weight of the chitosan was concluded to have no effect on the formation of chitosan–CNC PMC particles and only a minor effect on the shape and size of the particles. The higher turbidity levels for CNCs with lower sulfate group density and larger hydrodynamic diameter and for chitosans with higher DD were attributed to a larger number of CNCs being required for charge compensation.

## 1. Introduction

Chitosan is one of very few known polysaccharides that bear exclusively positive charges. A positive charge in polysaccharides is generally related to the amino group of 2-amino-2-deoxy-D-glucopyranosyl (GlcN) residues, which is protonated in acidic aqueous media. In addition to these residues, chitosan contains 2-acetamido-2-deoxy-D-glucopyranosyl (GlcNAc) residues, which under most conditions bear no charge. The proportions of GlcN and GlcNAc residues in a chitosan sample depend on the processing conditions during manufacture or preparation, involving the chemical or enzymatic deacetylation of the parent polysaccharide chitin. The fraction of GlcN residues in a chitosan sample is termed the degree of deacetylation (DD), generally expressed in percent. The polycationic nature and availability of chitosan render it attractive for applications involving ionic interactions. For such applications, the DD is a crucial parameter as it is related to the charge density along the polymer chain.

An area of intense research, into which chitosan has been absorbed, is the area of polyelectrolyte complexes (PECs). PECs are intermolecular complexes of oppositely charged polyelectrolytes based on attractive Coulomb interactions [1]. The interest in PECs is motivated by their numerous current and potential applications, such as the encapsulation of sensitive ingredients in food products [2], the delivery of drugs [3] and genes [4], the entrapment and delivery of proteins and immobilization of enzymes [5], and the encapsulation of cells [6]. The properties of PECs are governed by many factors, primarily the molecular weights and densities of ionizable groups of the two polyelectrolytes, the polyelectrolyte concentrations after mixing, the ratio of mixing, and the ionic strength and pH of the reaction medium.

The effect of molecular weight on the properties of PECs is incompletely understood. Several groups have reported the composition of PECs to be independent of the molecular weight of the polyelectrolytes [7,8]. Dautzenberg [9] and his collaborators have observed no effect of the molecular weight of sodium poly(styrene sulfate) on the structural parameters of its PECs with poly(diallyldimethylammonium chloride). De Vasconcelos et al. [10], on the other hand, have reported an increase and shift in the turbidity maximum in turbidimetric titrations of poly(methacrylic acid) (PMMA) with chitosan toward a higher chitosan/PMMA mass ratio with increasing molecular weight of PMMA. The authors concluded that PECs from PMMA of higher molecular weight were more water-soluble and larger than PECs from PMMA of lower molecular weight.

The effect of the density of ionizable groups, generally equated with the charge density, on the properties of PECs is much better understood as it has been more widely studied [7,11,12,13,14,15,16]. The results of these studies indicate that densely structured PECs are formed when the charge densities of the two polyelectrolytes are similar whereas highly swellable PECs with lower structural densities are formed in the case of a mismatch in the charge densities of the polyelectrolytes [13,17].

In previous studies [18,19,20], we investigated a new type of PEC between chitosan and cellulose nanocrystals (CNCs), which are anionic, rod-like nanoparticles. We referred to these complexes as polyelectrolyte–macroion complexes (PMCs) to emphasize the particulate nature of one of the two components. The properties and formation of these PMCs were governed by the large mismatch in density of the ionizable groups. A decrease in pH of the surrounding medium was found to increase the mismatch and cause PMC particle swelling whereas an increase in pH resulted in particle shrinking. Moreover, an increase in ionic strength caused a slight decrease in particles size, which was attributed to charge screening. Here we study the effects of chitosan molecular weight and DD on the preparation of chitosan–CNC PMC particles to test the hypothesis that their properties and formation can be controlled through these parameters. The impact of CNC properties on PMC formation was also assessed.

## 2. Results

Three commercial chitosan samples with different molecular weight specifications (high, medium, and low) were characterized according to molecular weight, degree of deacetylation, hydrodynamic diameter, electrophoretic mobility, and amino group density. The characteristics of the three chitosan samples are listed in Table 1.

CNCs with two different sulfate group densities, denoted CNC-18 and CNC-33, were prepared by sulfuric acid hydrolysis of a dissolving-grade softwood sulfite pulp and characterized according to hydrodynamic diameter, electrophoretic mobility, and sulfate group density. The characteristics of the two CNC samples are listed in Table 2.

### 2.1. Effect of Chitosan Molecular Weight

**Figure 1:**
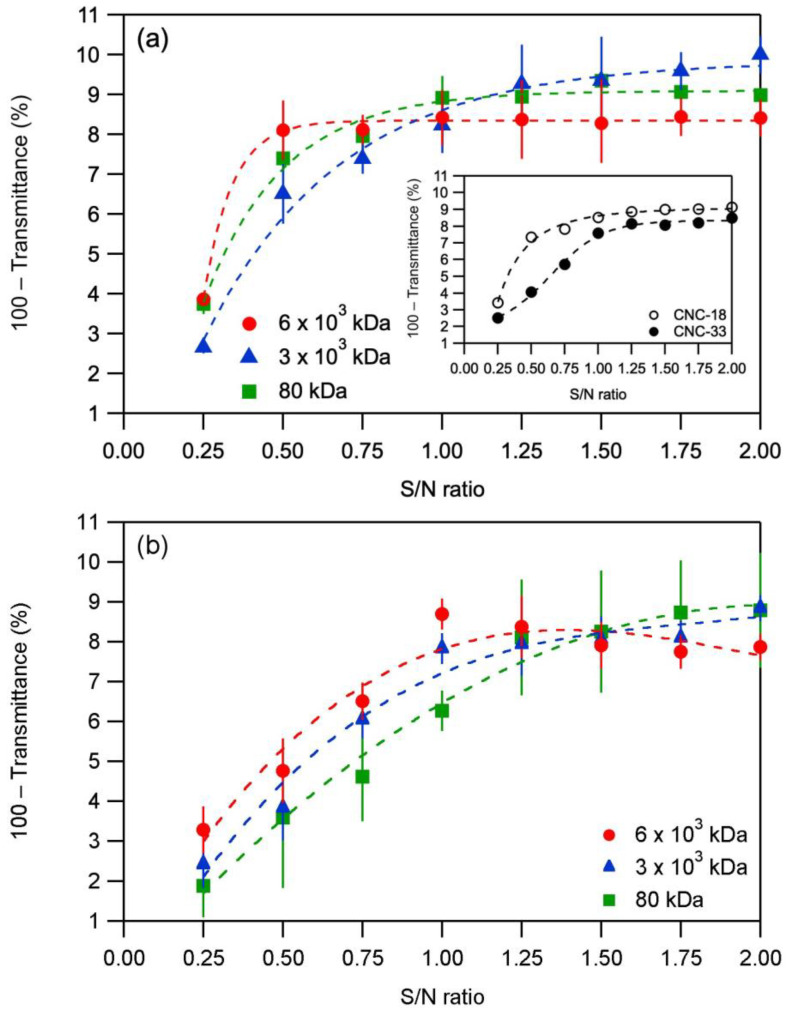
Turbidity curves for titrations of solutions of chitosans with different molecular weights with (**a**) CNC-18 suspensions and (**b**) CNC-33 suspensions. (The dashed lines are merely visual guides. Each data point is a mean of three measurements. The error bars correspond to ± one standard deviation.) The inset shows the averaged data for CNC-18 and CNC-33. S/N ratio = number of CNC sulfate groups divided by number of chitosan amino groups, 100 − Transmittance of deionized water = 0%.

The complexation of CNCs with chitosans of different molecular weights was analyzed by turbidimetric titration. The turbidity curves for titrations of chitosan solutions with suspensions of CNC-18 and CNC-33 are shown in Figure 1. In all titrations, on addition of the CNC suspension to the chitosan solution, the turbidity increased rapidly initially and then leveled off or decreased slightly at higher S/N ratios. The titration curves for different molecular weights of chitosan differed slightly. Because of the large standard deviations of the measured turbidity values (error bars in Figure 1), the significance of the observed differences in the titration curves was assessed by one-way ANOVA of the turbidity data. The ANOVA test results are listed in Table 3. Statistical analysis of the turbidity data revealed that there was no statistically significant difference between the data from different molecular weight chitosans in both cases, titration with CNC-18 and with CNC-33. Thus, an effect of the molecular weight of the chitosan was not observed in the turbidimetric titrations experiments.

Our results are in accordance with those studies that have found that the molecular weight to have no effect on the parameters of PMCs [7,8,9].

Averaging of the turbidity levels observed with different chitosan molecular weights (inset in Figure 1a), showed that on average, CNC-18 caused higher turbidity levels than CNC-33 at any given S/N ratio. The higher turbidity levels are consistent with a higher cellulose/chitosan mass ratio required for charge stoichiometry in the case of CNC-18, with a lower sulfate group density. At a pH of 2.6, used in these titrations, the degree of ionization of the CNCs was 0.58 (p*K*_a_ of 2.46 [19]), i.e., 58% of the available sulfate groups were deprotonated and negatively charged. Charge stoichiometry, or an NH_3_^+^/SO_3_^−^ ratio of unity, required a cellulose/chitosan mass ratio of about 57:1 for CNC-18 and of about 30:1 for the CNC-33. As a result, PMC particles prepared with CNC-18 probably contained more (and larger) CNCs and were bulkier than PMC particles prepared with CNC-33. The more rapid formation and bulkier nature of the PMC particles formed by CNC-18 might be the reason for the observed faster increase in turbidity.

**Figure 2:**
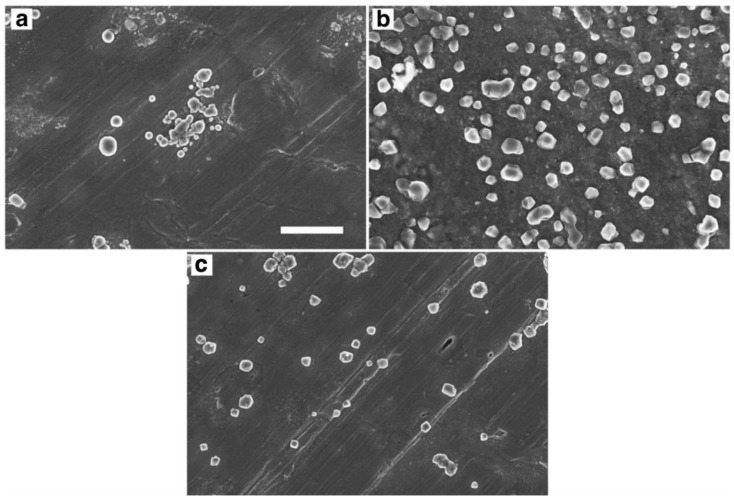
Field emission scanning electron microscopy images of chitosan–cellulose nanocrystal (CNC) polyelectrolyte–macroion complex particles prepared by addition of a CNC-18 suspension to solutions of chitosan with different molecular weights: (**a**) 6 · 10^3^ kDa, (**b**) 3 · 10^3^ kDa, (**c**) 80 kDa. Scale bar (applying to all images): 3 μm.

The effect of chitosan molecular weight on the morphology of chitosan–CNC PMC particles was studied by SEM. FE-SEM images of PMC particles prepared with CNC-18 and different molecular weight chitosans are shown in Figure 2. The particles from high-molecular-weight chitosan appeared to be more spherical than the particles from medium and low molecular weight chitosans. This finding is in accordance with the results of Ko et al. [21], who reported more spherical particle shapes at higher molecular weights for tripolyphosphate-crosslinked chitosan microparticles.

Table 4 lists the electrophoretic mobilities and hydrodynamic diameters of chitosan–CNC PMC particles from different molecular weight chitosans. The particles were formed at an N/S ratio of 0.3 to yield sizes within the analytical range, i.e., particles formed at a higher N/S ratio exceeded the size limit of the instrument. The particles from medium-molecular-weight chitosan were slightly larger than those from high and low-molecular weight chitosan. PMC particles from the latter two types were of comparable size. The slightly larger size of the PMC particles from medium-molecular-weight chitosan might indicate an optimum length ratio of the PMC components. The estimated length ratios (contour length of the chitosan molecules/length of the CNCs) were 150, 80, and 2:1 for the high, medium, and low-molecular weight chitosan, respectively, using 120 nm as an average CNC length. The electrophoretic mobilities of the PMC particles were positive for all chitosan types, indicating a non-stoichiometric composition and excess of ammonium groups in the particles.

### 2.2. Effect of Chitosan Degree of Deacetylation

The effect of the DD of chitosan on chitosan–CNC PMC particles was studied by turbidimetric titration using chitosan samples subjected to a chitin deacetylation procedure. The characteristics of these chitosan samples are listed in Table 5. The data in Table 5 shows that the deacetylation procedure caused a decrease in the molecular weight in addition to an increase in the DD.

**Figure 3:**
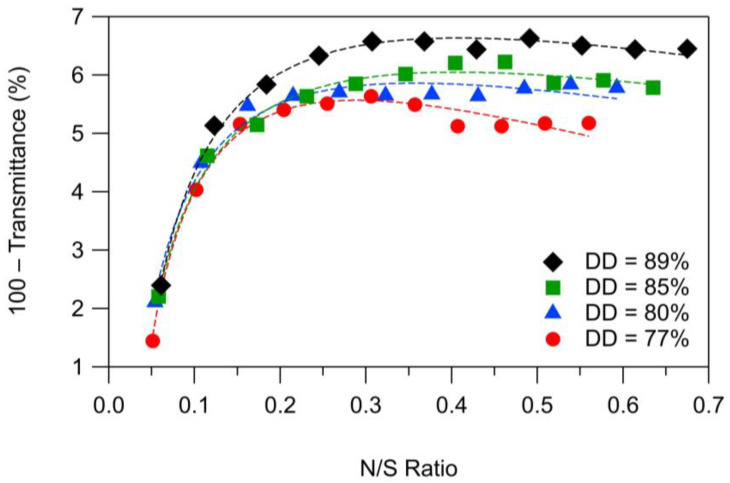
Titration curves for turbidimetric titrations of CNC-18 suspensions with solutions of chitosans with DDs ranging from 77 to 89%. (The dashed lines are merely visual guides. Each data point is a mean of three measurements. Error bars are omitted for clarity.) N/S ratio = number of chitosan amino groups divided by number of CNC sulfate groups, 100 − Transmittance of deionized water = 0%.

The turbidity curves for titrations of CNC-18 suspensions with chitosan solutions of different DD are shown in Figure 3. Upon addition of the chitosan solution to the CNC suspension, the turbidity increased rapidly initially and then leveled off. At higher volumes of chitosan solution added, the turbidity decreased slightly in accordance with the results reported in Wang and Roman [18] for the effect of mixing sequence.

Overall, the turbidity at any given N/S ratio was higher for higher DD values. To establish whether the observed effect was related to the sample differences in DD or molecular weight, we analyzed the turbidity data by one-way ANOVA. The ANOVA test results are shown in Table 6.

Analysis of the data by one-way ANOVA showed that the molecular weight did not have a statistically significant effect on the turbidity and that the differences in the turbidity curves were due to the differences in DD. Post hoc comparisons using the Tukey HSD (Honestly Significant Difference) test (Table 7) indicated that the turbidity levels for a DD of 80 and 77% were statistically significantly lower than for a DD of 89 and 85%.

A higher DD signifies a higher charge density, requiring a larger number of CNCs for charge compensation. Thus, PMC particles from chitosans with higher DDs might contain a larger number of CNCs and therefore be larger than PMC particles from chitosans with lower DDs.

## 3. Discussion

Chitosan and cellulose are both natural polysaccharides with good biodegradation and biocompatibility profiles. CNCs prepared by sulfuric acid hydrolysis are negatively charged because they undergo partial surface esterification during the hydrolysis process, resulting in highly acidic sulfate groups [22]. Chitosan carries amino groups, resulting from the deacetylation of acetamido groups that are protonated and positively charged at acidic pH levels [23]. Upon mixing, the negatively charged CNCs and positively charged chitosan molecules aggregate and form well-defined PMC particles. These PMC particles have a strong potential for applications in agriculture and oral drug delivery, where their biodegradability and biocompatibility are important attributes. Different applications will require different PMC properties, making a detailed understanding of the factors that govern their formation and properties essential.

The present study is the third in a series of studies investigating the effects of process parameters and chitosan properties on the formation and properties of chitosan–CNC PMC particles [20]. The first study [18] focused on the effects of the sequence of mixing of a chitosan solution and a CNC suspension as well as the CNC concentration. The study revealed that when a CNC suspension is added to a chitosan solution, the turbidity increases and then levels off, whereas when a chitosan solution is added to a CNC suspension, the turbidity reaches a maximum and then decreases on further chitosan addition, suggesting a partial dissociation of aggregates due to charge overcompensation. The study also showed that chitosan–CNC PMC particles are composed primarily of CNCs, due to the lower charge density of CNCs (0.18–0.33 versus 5.3–6.1 mol/kg), and range in size from a few hundred nanometers to several micrometers, depending on the cellulose/chitosan ratio. Interestingly, particles formed at amino/sulfate group molar ratios >1, carrying a positive charge, had more or less spherical shapes, whereas particles formed at ratios <1, carrying a negative charge, had well-defined polygonal shapes.

The second study [19] investigated the effects of pH and salt concentration on the formation and properties of chitosan–CNC PMC particles. The p*K*_b_ of chitosan was measured by potentiometric titration as 6.40 ± 0.03. The p*K*_a_ of the CNCs was determined through extrapolation of a degree of ionization vs. pH curve as 2.46 ± 0.12. The pH range in which both the CNCs and chitosan were completely ionized was found to be narrow and centered at 4.5. A pH increase from this range was observed to cause a turbidity decrease due to shrinking of the PMC particles on a decrease in the degree of ionization of the chitosan chains. A pH decrease, on the other hand, resulted in a steep increase in turbidity, which was attributed to an increase in particles size as a result of a decrease in the degree of ionization of the CNCs. The size changes in the PMC particles were confirmed by SEM. A decrease in turbidity was also observed on an increase in salt concentration and was shown to be attributable to charge-screening-related shrinking of the PMC particles.

This study investigated two additional variables that may be expected to affect the formation and properties of chitosan–CNC PMC particles, namely chitosan molecular weight and DD. The results of this study complete the picture by revealing that chitosan molecular weight has no apparent effect on the formation of the PMC particles and only a minor, if any, effect on their shape and size. The observed effect of DD, specifically higher DDs resulting in higher turbidity levels, is consistent with the previously reported increase in PMC hydrodynamic diameter with increasing amino/sulfate group molar ratio, observed when titrating a CNC suspension with a chitosan solution [18], as well as the previously observed increase in turbidity after lowering of the pH from 4.5, causing a decrease of CNC degree of ionization [19].

In summary, the three studies allow the following conclusions:

- Chitosan–CNC PMC particles consist primarily of CNCs held together by (less bulky) chitosan molecules.
- PMC particle size can be adjusted from a few hundred nanometers to several micrometers and net particle charge from negative to positive by controlling the amino/sulfate group molar ratio through selection of chitosan DD and chitosan-to-CNC mass ratio. (The charge density of CNCs is low, compared to the charge density of chitosan, and controllable only within limits).
- In environments with a pH below 2, chitosan–CNC PMC particles will be larger, and in environments with a pH above 7, smaller, than in environments with a pH between 2 and 7. This behavior is particularly interesting for the oral delivery of therapeutics and nutraceuticals.
- In environments with a salt concentration above 0.1 M, chitosan–CNC PMC particles will be smaller and more compact than in environments with a salt concentration below 0.1 M.

## 4. Materials and Methods

### 4.1. Materials

High, medium, and low molecular weight chitosans (Fluka BioChemika) were purchased from Sigma-Aldrich (St. Louis, MO, USA). Chitin was purchased from ACROS Organics (Morris Plains, New Jersey, USA). NaCl (certified), HCl (0.1 N, both certified), H_2_SO_4_ (>95%), and NaOH (0.1 N and 1.0 N, certified) were obtained from Fisher Scientific (Waltham, MA, USA). 50% NaOH was ACS reagent grade and purchased from Ricca Chemical Company (Arlington, TX, USA). All experiments were conducted with deionized water (resistivity at 25 °C: 18.2 MΩ·cm) prepared with a Direct-Q 5 Ultrapure Water System (Millipore, Burlington, MA, USA).

### 4.2. CNC Preparation

The method of preparation of the CNCs was described in Wang and Roman [18]. Briefly, a 50 g portion of softwood dissolving pulp (Temalfa 93 A-A, sulfite pulp), generously donated by Tembec, Inc. (Témiscaming, QC, Canada) was ground to pass a 60-mesh screen. The obtained powder was added to 500 mL of 64 wt. % H_2_SO_4_, preheated to 45 °C, and hydrolyzed under stirring at that temperature. After 45 min, the reaction medium was poured into 4.5 L of deionized water after which the CNCs were isolated by centrifugation and purified by dialysis with deionized water. When the pH of the dialysis water remained unchanged, the dialysis was discontinued. The suspension was then cooled with an ice-bath for sonication and filtered sequentially through 1, 0.45, and 0.22 μm Millipore polyvinylidene fluoride (PVDF) syringe filters. After filtration, the CNC stock suspension generally had a concentration between 0.6 and 0.9% (*w*/*v*). To determine the effects of CNC properties, a second CNC sample was prepared with a hydrolysis time of 60 min instead of 45 min. The CNCs were characterized as described in Wang and Roman [18].

### 4.3. Purification of Commerical Chitosans

For purification, 1 g of commercial chitosan was added to 250 mL 0.1 N HCl and allowed to dissolve overnight. Next, the solution was filtered sequentially through 1, 0.45, and 0.22 μm PVDF syringe filters. Following filtration, 1 N NaOH was added to the solution until its pH was between 9 and 10. After isolation by centrifugation at 4 °C and 4900 rpm for 15 min, the precipitated chitosan was further purified by washing thrice with deionized water, followed by freeze-drying.

### 4.4. Preparation of Chitosan Samples with Different DDs

Chitosan samples with different DDs were prepared by deacetylation of chitin according to literature procedures [24,25]. In detail, 20 g chitin was heated for 1 h under stirring in 400 mL 47% NaOH at 110 °C in a nitrogen atmosphere. The reaction mixture was allowed to cool to 80 °C and the reaction product was washed with deionized water and dried for 2 h in an oven set to 105 °C. Approximately 5 g of the reaction product was set aside, and the rest was subjected again to the procedure above. This process was repeated twice more to yield a total of four samples with different DDs. Finally, the chitosan samples were purified as described above by dissolution, filtration, precipitation, washing, and freeze-drying.

### 4.5. Chitosan Characterization

#### 4.5.1. Molecular Weight

The molecular weights of the four prepared chitosan samples were determined as described in Wang and Roman [18] using the viscosity method that is based on the Mark–Houwink equation:

[η] = *KM^a^* (1)

where [η] is the intrinsic viscosity, *M* is the viscosity-average molecular weight, and *K* and *a* are the Mark–Houwink parameters. Viscosity measurements were performed at 25 °C using an Ubbelohde capillary viscometer with an inner capillary diameter of 0.53 mm and chitosan solutions in the binary solvent system 0.3 M acetic acid/0.2 M sodium acetate [26], ranging in concentration from 0.1 to 1 mg/mL. The values used for the Mark–Houwink parameters were *a* = −1.02 · 10^−2^ · DD + 1.82 and *K* = 1.64 · 10^−30^ · DD^14.0^ [27].

#### 4.5.2. Degree of Deacetylation

The DDs of the commercial chitosans were determined by ^1^H-NMR spectroscopy as described in Wang and Roman [18]. Briefly, chitosan was dissolved in a mixture of D_2_O and DCl, and spectra were acquired at 90 ± 1 °C using a Varian Unity 400 MHz NMR spectrometer (Varian, Inc., Palo Alto, CA, USA). The DD was calculated as

(2) $$\mathrm{DD}=\left[\frac{H1D}{\left(H1D+HAc/3\right)}\right]\times 100\%$$

where *H*1*D* is the integral of the H1 GlcN peak and *HAc* is the integral of the peak corresponding to the three protons of the GlcNAc acetyl group [28].

The DDs of the four chitosan samples obtained by the deacetylation of chitin were determined by FTIR spectroscopy according to the method by Baxter et al. [29], which was chosen for its greater convenience in combination with an equivalent accuracy, compared to the NMR-based method above. The method is based on the intensity ratio of the amide I band at 1650 cm^−1^ and the OH band at 3450 cm^−1^. FTIR spectra were recorded from KBr pellets, prepared as follows. Ninety-eight milligrams of dry KBr were ground in a mortar with 2 mg of sample. The mixture was transferred into a deep stainless-steel nut, with a stainless-steel bolt inserted from one end. The KBr pellet was generated by inserting a second bolt from the opposite end and compressing the powder for a period of about 10 min. Before data collection, the nut with the KBr pellet was placed in a vacuum oven for sample drying. FTIR spectra were recorded with a Thermo Nicolet Nexus 470 FTIR spectrometer (Thermo Fisher Scientific, Inc., Waltham, MA, USA) using 128 as the number of scans and 4 cm^−1^ as the spectral resolution.

#### 4.5.3. Hydrodynamic Diameter and Electrophoretic Mobility

The z-average hydrodynamic diameter (cumulants mean) and electrophoretic mobility of the chitosan samples were measured in triplicate at 25 °C using a 0.001% (*w*/*v*) chitosan solution and Malvern Zetasizer Nano ZS with Malvern DTS1060 folded capillary cells (Malvern Instruments, Inc., Westborough, MA, USA) without adjustment of the pH or ionic strength.

#### 4.5.4. Amino Group Density

The amino group densities of the commercial chitosans were determined through conductometric titration using a Mettler Toledo SevenMulti S47 pH/conductivity meter equipped with an InLab 730 conductivity probe (Mettler-Toledo Inc., Columbus, OH, USA). A 0.1N NaOH solution was added dropwise under stirring and N_2_ to 25 mL of a 0.1% (*w*/*v*) chitosan solution with an NaCl concentration of 0.1 M. The conductivity was recorded after every 10 drops and the amino group density calculated from the titrant volume between the two equivalence points. Measurements were performed in triplicate.

### 4.6. Preparation of Dilute Chitosan Solutions

After drying in an oven for 2 h at 105 °C, a 0.1 g amount of purified chitosan was added to 100 mL of 0.1 N HCl to give a ~0.1% (*w*/*v*) chitosan stock solution. The stock solution’s concentration was measured by thermogravimetric analysis and calculated by averaging three measurements. Further details are provided in Wang and Roman [18]. Starting solutions for the complexation experiments were obtained by dilution of the stock solution with deionized water. Prior to the complexation experiments, 0.1 N HCl or 0.1 N NaOH and NaCl were added to the solutions until the desired pH and ionic strength, respectively, were reached.

### 4.7. Preparation of Dilute CNC Suspensions

Starting suspensions for the complexation experiments were obtained by dilution of the filtered stock suspension with deionized water. Prior to the complexation experiments, 0.1 N HCl or 0.1 N NaOH and NaCl were added to the suspensions until the desired pH and ionic strength, respectively, were reached.

### 4.8. Turbidimetric Titrations

In a turbidimetric titration, a chitosan solution (0.001% (*w*/*v*)) was added to a CNC suspension (0.02% (*w*/*v*)), or vice versa. Prior to the experiment, the ionic strength and pH of the chitosan solution and CNC suspension were adjusted to 1 mM and 2.6, respectively. Titrations were performed by drop-wise addition under vigorous agitations with a magnetic stir bar. A probe colorimeter (PC 950, Brinkmann Instruments, Inc., Westbury, NY, USA), operating at a wavelength of 420 nm and with an optical cell with a 2 cm path length, was used for measuring the transmittance of the reaction mixture. Turbidity values are reported as 100 − transmittance (%). Each experiment was performed after zeroing of the probe colorimeter in deionized water and was repeated two times.

### 4.9. Characterization of Chitosan–CNC PMC Particles

#### 4.9.1. Morphology

The morphology of chitosan–CNC PMC particles was investigated with a LEO 1550 field emission scanning electron microscope (LEO Electron Microscopy, Inc., Thornwood, NY, USA). A working distance of 4 mm and an accelerating voltage of 5 kV were used for FE-SEM imaging. Samples were prepared by mixing of a CNC suspension (0.02% (*w*/*v*)) and a chitosan solution (0.001% (*w*/*v*)), each with an ionic strength of 1 mM and a pH of 3.4, under strong agitation with a magnetic stir bar. The volumes of the two liquids were chosen to give an amino/sulfate group molar ratio (N/S ratio) of 1. Drops of 10 μL of the suspensions, containing PMC particles, were placed onto conductive Ni–Cu tape (Ted Pella) on standard SEM stubs (Ted Pella). After drying of the droplets under ambient conditions, a thin (~6 nm) layer of carbon was deposited onto the samples.

#### 4.9.2. Hydrodynamic Diameter and Electrophoretic Mobility

The electrophoretic mobility and z-average hydrodynamic diameter of chitosan–CNC PMC particles were measured by laser Doppler electrophoresis in a Malvern Zetasizer Nano ZS90 (Malvern Instruments, Inc., Westborough, MA, USA). Samples were prepared by mixing of a CNC suspension (0.02% (*w*/*v*)) and a chitosan solution (0.001% (*w*/*v*)), each with an ionic strength of 1 mM and a pH of 2.6, under strong agitation with a magnetic stir bar. The volumes of the two liquids were chosen to give sulfate/amino group molar ratio (S/N ratio) of 0.3. The obtained PMC particle suspensions were used without further dilution or filtration. Each measurement was performed at 25 °C in a Malvern DTS1060 folded capillary cell and was repeated two times.

### 4.10. Statistical Data Analysis

The turbidity data from the turbidimetric titrations were analyzed by one-way analysis of variance (ANOVA) with a 95% confidence interval (α = 0.05) and Tukey’s HSD test. Statistical analysis of the data was carried out with the SAS software JMP 7.0.2.

## 5. Conclusions

The effects of chitosan molecular weight and DD on the formation and properties of chitosan–CNC PMC particles were investigated to assess the impact of these parameters. The molecular weight of the chitosan appeared to have no apparent effect on the formation of chitosan–CNC PMC particles and only a minor, if any, effect on the shape and size of the particles. The sulfate group density of CNCs showed an inverse relationship with turbidity levels, likely due to a larger number of CNCs required for charge compensation at lower sulfate group densities. Similarly, chitosans with higher DDs gave higher turbidity levels, attributable to the higher chitosan charge density requiring a larger number of CNC for charge compensation.

## Figures and Tables

**Table 1:** Characteristics of the high, medium, and low molecular weight chitosans.

Chitosan	Molecular Weight ^a^ (kDa)	DD ^b^ (%)	Hydrodynamic Diameter ^c^ (nm)	Electrophoretic Mobility ^d^ (μm·cm/V·s)	Amino Group Density ^e^ (mol/kg)
High Mw ^f^	6 · 10^3^	87	319 ± 18	2.6 ± 0.2	6.1
Medium Mw ^f^	3 · 10^3^	88	265 ± 14	3.2 ± 0.1	5.8
Low Mw ^f^	81	88	238 ± 52	1.7 ± 0.9	5.3

^a^ Calculated from the intrinsic viscosity; ^b^ degree of deacetylation determined by ^1^H NMR; ^c^ Determined by dynamic light scattering; ^d^ Measured with a Malvern Zetasizer Nano ZS 90; ^e^ Determined by conductometric titration; ^f^ molecular weight.

**Table 2:** Characteristics of the cellulose nanocrystal (CNC) samples.

CNC Sample	Hydrodynamic Diameter ^a^ (nm)	Electrophoretic Mobility ^b^ (μm·cm/V·s)	Sulfate Group Density ^c^ (mol/kg)
CNC-18	104 ± 4	−2.8 ± 0.2	0.18
CNC-33	68 ± 5	−3.1 ± 0.4	0.33

^a^ Determined by dynamic light scattering; ^b^ Measured with a Malvern Zetasizer Nano ZS 90; ^c^ Determined by conductometric titration.

**Table 3:** One-way ANOVA test results (α = 0.05) for the effect of molecular weight of the commercial chitosan samples on the turbidity.

Source of CNCs ^a^	DF ^b^	F Ratio ^c^	Prob > F ^d^
Batch 1	2	0.1250	0.8827
Batch 2	2	0.4208	0.6580

^a^ Cellulose nanocrystals; ^b^ degrees of freedom; ^c^ mean of squares between groups divided by mean of squares within groups; ^d^ null hypothesis: all groups for the study are the same, rejected for Prob > F values larger than α.

**Table 4:** Hydrodynamic diameter and electrophoretic mobility of polyelectrolyte–macroion comples (PMC) particles from different molecular weight chitosans.

Sample	Hydrodynamic Diameter (nm)	Electrophoretic Mobility (μm·cm/V·s)
PMC with high-molecular-weight chitosan	373 ± 9	3.0 ± 0.1
PMC with medium-molecular-weight chitosan	539 ± 32	3.4 ± 0.1
PMC with low-molecular-weight chitosan	363 ± 37	2.9 ± 0.2

**Table 5:** Characteristics of the chitosan samples prepared in-house by chitin deacetylation.

Number of Deacetylation Cycles	Molecular Weight ^a^ (kDa)	DD ^b^ (%)
1	1.7	77
2	1.9	80
3	1.4	85
4	1.0	89

^a^ Calculated from intrinsic viscosity; ^b^ Degree of deacetylation determined by FTIR spectroscopy.

**Table 6:** One-way ANOVA test results (α = 0.05) for the effects of DD and molecular weight of the deacetylated chitin samples on the turbidity.

Effect	DF ^b^	F Ratio ^c^	Prob > F ^d^
Molecular weight	2	1.7644	0.2497
DD ^a^	3	8.7221	<0.0001 *

^a^ degree of deacetylation; ^b^ degrees of freedom; ^c^ means of squares between groups divided by mean of squares within groups; ^d^ null hypothesis: all groups for the study are the same, rejected for Prob > F values larger than α; * indicates rejection of the null hypothesis.

**Table 7:** Significance levels and least squares mean (Tukey HSD, α = 0.05) for the effect of degree of deacetylation (DD) of the deacetylated chitin samples on the turbidity.

Number of Deacetylation Cycles	Level (DD %)	Significance ^a^	Least Squares Mean
1	77	A	4.87
2	80	A	5.27
3	85	B	5.42
4	89	B	5.98

^a^ Levels not connected by same letter are statistically significantly different.

## Data Availability

Reported data are available upon request. Inquiries should be directed to the corresponding author.

## Sample Availability

There are no available samples.
